# ILK supports RhoA/ROCK-mediated contractility of human intestinal epithelial crypt cells by inducing the fibrillogenesis of endogenous soluble fibronectin during the spreading process

**DOI:** 10.1186/s12860-020-00259-0

**Published:** 2020-03-17

**Authors:** David Gagné, Yannick D. Benoit, Jean-François Groulx, Pierre H. Vachon, Jean-François Beaulieu

**Affiliations:** 1 Laboratory of Intestinal Physiopathology, Faculty of Medicine and Health Sciences, Université de Sherbrooke, and Research Center of the Centre Hospitalier Universitaire de Sherbrooke, Sherbrooke, QC, J1H 5N4 Canada; 2 Department of Cellular and Molecular Medicine, University of Ottawa, Ottawa, ON K1H 8M5 Canada; 3 Section of Cell and Developmental Biology, Division of Biological Sciences, University of California San Diego, La Jolla, California, 92093 USA; 4 Department of Anatomy and Cell Biology, Faculty of Medicine and Health Sciences, Université de Sherbrooke, Sherbrooke, Québec, J1H 5N4 Canada

**Keywords:** ILK, IPP complex, Integrin, α5β1, RhoA, Fibronectin, Fibrillogenesis, Actin stress fibers, Cell contractility, Epithelial cells

## Abstract

**Background:**

Fibronectin (FN) assembly into an insoluble fibrillar matrix is a crucial step in many cell responses to extracellular matrix (ECM) properties, especially with regards to the integrin-related mechanosensitive signaling pathway. We have previously reported that the silencing of expression of integrin-linked kinase (ILK) in human intestinal epithelial crypt (HIEC) cells causes significant reductions in proliferation and spreading through concomitantly acquired impairment of soluble FN deposition. These defects in ILK-depleted cells are rescued by growth on exogenous FN. In the present study we investigated the contribution of ILK in the fibrillogenesis of FN and its relation to integrin-actin axis signaling and organization.

**Results:**

We show that de novo fibrillogenesis of endogenous soluble FN is ILK-dependent. This function seemingly induces the assembly of an ECM that supports increased cytoskeletal tension and the development of a fully spread contractile cell phenotype. We observed that HIEC cell adhesion to exogenous FN or collagen-I (Col-I) is sufficient to restore fibrillogenesis of endogenous FN in ILK-depleted cells. We also found that optimal engagement of the Ras homolog gene family member A (RhoA) GTPase/Rho-associated kinase (ROCK-1, ROCK-2)/myosin light chain (MLC) pathway, actin ventral stress fiber formation, and integrin adhesion complex (IAC) maturation rely primarily upon the cell’s capacity to execute FN fibrillogenesis, independent of any significant ILK input. Lastly, we confirm the integrin α5β1 as the main integrin responsible for FN assembly, although in ILK-depleted cells αV-class integrins expression is needed to allow the rescue of FN fibrillogenesis on exogenous substrate.

**Conclusion:**

Our study demonstrates that ILK specifically induces the initiation of FN fibrillogenesis during cell spreading, which promotes RhoA/ROCK-dependent cell contractility and maturation of the integrin-actin axis structures. However, the fibrillogenesis process and its downstream effect on RhoA signaling, cell contractility and spreading are ILK-independent in human intestinal epithelial crypt cells.

## Background

ECM constituents such as FN are bound principally by heterodimeric integrin receptors [1, 2]. The binding of integrins to their specific ECM ligands induces clustering of the former and the recruitment of various types of proteins constituting the integrin adhesome, including several intracellular adaptors/scaffolders and signaling proteins such as talin, kindlin, vinculin, paxillin, ILK tensin, focal adhesion kinase (FAK) and Src protein-tyrosine kinase [1].

Integrin adhesion complexes (IAC) act as critical physical links between the ECM and the actin-based cytoskeleton (e.g. stress fibers), in addition to constituting functional cellular mechanosensing centers linked to the intracellular signaling network (e.g. RhoGTPases), which in turn direct cell response to ECM properties (e.g. stiffness, molecular composition, and spacing) [1–4]. Three major types of IAC linked to the actin cytoskeleton are usually defined in 2D cell culture, namely focal complexes (FX), focal adhesions (FA) and fibrillar adhesions (FB) [5, 6]. FX originate from nascent integrin adhesion sites and are typically small, punctuate structures formed at the edges of lamellipodia [6]. As the cell edge progress with cycles of lamellipodial protrusion-retraction and matrix testing in spreading and migrating cells [4–6], developing tensile force applied by the actomyosin contractile machinery leads to additional recruitment of adhesome components and stabilization of some FX into FA, the latter thereafter can further mature into larger FA in the innermost areas of a cell’s lamellipodia [5, 6]. Eventually, force applied by stress fibers anchor to FA help to sequestrate tensin and integrin α5β1 centripetally to form elongated fibrillar structures [7], thus constituting the defining step in the formation of FB [5, 8].

The stimulation of the RhoA/ROCK pathway, which leads to phosphorylation of the S19 residue of MLC and activation of myosin II motor function, is central to actomyosin tension-driven assembly of FA and stress fibers [6, 9]. At least four distinct subtypes of stress fibers that form interrelated networks have been identified in adherent mammalian cells [9, 10]. The non-contractile dorsal stress fiber has one-end anchored to FA and forms orthogonal networks with coupled contractile arc transverses. The highly contractile ventral stress fibers are attached at both ends to FA and typically arise from the fusion and reorganization of the two previous types. Accordingly, the assembly of multiple parallel ventral stress fibers – and likewise of contractile actin cap fibers – constitutes a major indicator of muscle and non-muscle cell’s polarized “contractile phenotype” [9–11].

The formation of FB is closely associated with the assembly of FN into the ECM. In this process, called FN fibrillogenesis, intracellular actomyosin tension transmitted to integrin α5β1 unfolds soluble FN dimers to expose cryptic interaction sites required to initiate FN auto-assembly and multimerization into fibrils [8, 12]. These fibrils are in turn integrated into insoluble fibers to form a mature ECM fibrillar network, alongside collagens. The RGD sequence of the FNIII_10_ domain of FN serves as the primary interaction site for integrin binding at the cell surface. Integrin α5β1 uses both the RGD site and the adjacent FNIII_9_ domain PHSRN (synergy) site that is required for the selective binding and assembly of soluble FN [8, 13], as well as form a “tensioned” catch bond and reinforce binding to FN [14, 15]. Incidentally, the αVβ3 integrin is another FN receptor that shows catch bond behavior [3] and which under some conditions can conduce FN assembly [16–18]. Both α5β1 and αV-class integrin-mediated adhesion to FN contribute to the regulation of RhoGTPases and mechanotransduction [3, 19]. However, studies suggest that αVβ3 supports peripheral FA adhesion [20], whereas α5β1 supports RhoA coupling to ROCK/MLC pathway [20] and additional strengthening of cell adhesion [21, 22].

ILK is another player of note in the integrin adhesome [1, 23, 24]. This pseudokinase binds parvin (α, β, or γ) at its C-terminal, as well as PINCH (− 1 or − 2) at its N-terminal, subsequently forming the heterotrimeric ILK/PINCH/parvin (IPP) complex. The IPP complex is a major constituent of the β1 and β3 integrin adhesome [1, 24], acting as a multi-domain scaffolder that promotes integrin-actin linkage stabilization, actin filaments (F-actin) bundling and FN/ECM deposition [23–25]. In a previous study, we showed that in the human intestinal mucosa, the members of the IPP complex are mainly expressed at the basolateral membrane of the proliferative epithelial cells of the crypt compartment, closely following the distribution of FN in the underlying ECM basal membrane [26]. We also reported that the silencing of ILK expression in HIEC cells results in a concomitant loss of other IPP complexes proteins, as well as significant reductions of FN deposition, proliferation and migration capacities [26].

In the present study, considering the importance of FN for the intestinal crypt ECM/niche [27] and that ILK contribution to the regulation of intestinal epithelial cell functions (e.g. proliferation, restitution and wound healing) is related to FN deposition or adhesion [26, 28–31], we further investigated the functional linkage between ILK and FN for the regulation of integrin-actin axis dynamics in the context of HIEC cell spreading.

## Results

### ILK-silencing results in altered actin cytoskeletal organization and morphology in HIEC cells

**Fig. 1:**
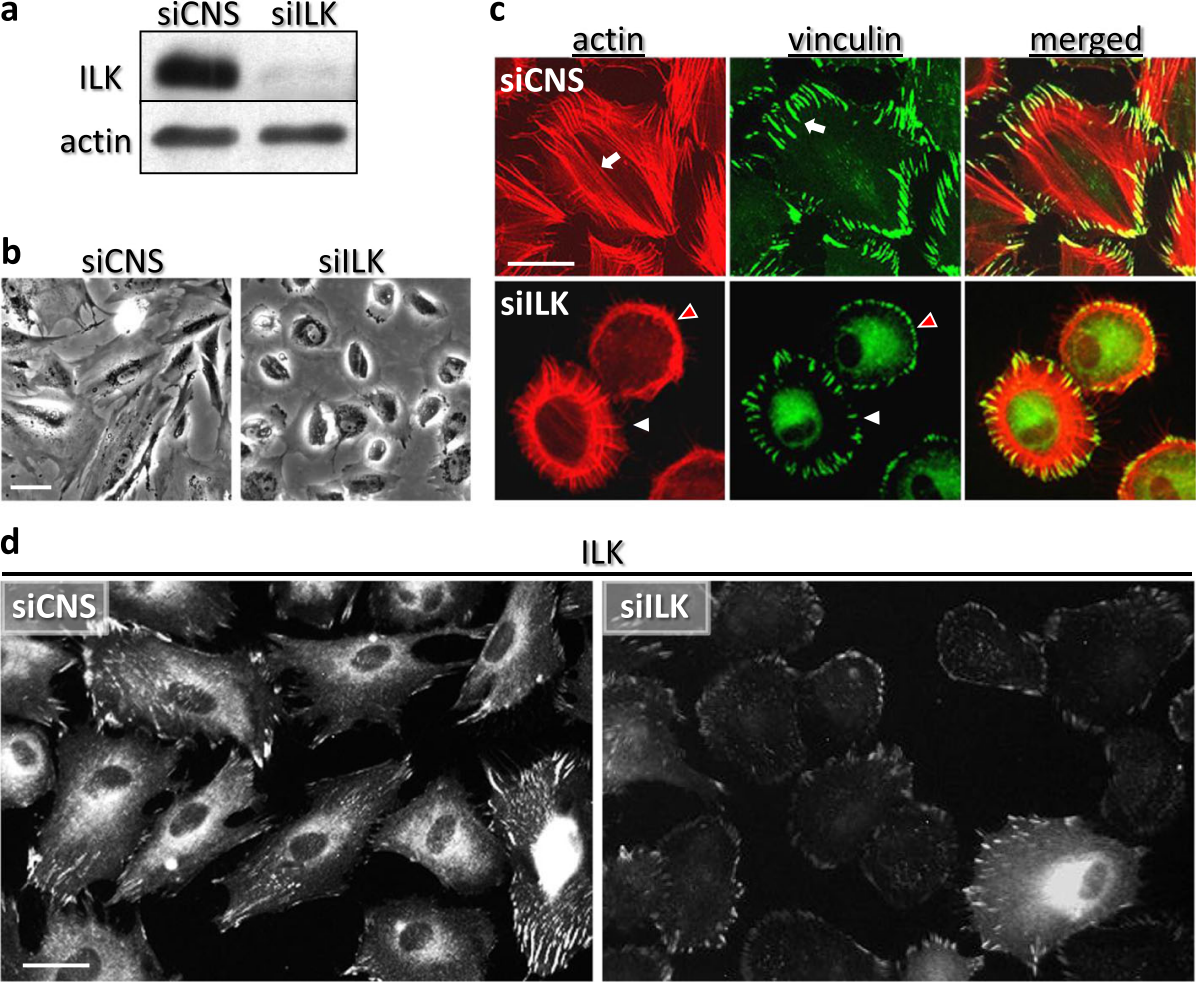
Effect of ILK silencing on structural and morphological characteristics of HIEC cells. (a to d) HIEC were transfected with 20 nM of control, non-interfering siRNA (siCNS) or an siRNA directed against ILK expression (siILK). **a** Western blot (WB) analysis of ILK protein levels in whole-cell lysates of siCNS and siILK cells 48 h after the transfection. β-actin protein levels were used as the loading control. **b** Inverted contrast microscopy images of siCNS and siILK cells grown on plastic dishes. **c** Immunofluorescence (IF) microscopy images of the actin cytoskeleton (TRITC-phalloidin; red) and vinculin (anti-vinculin antibody; green) staining in siCNS and siILK cells grown for 24 h on serum-pretreated glass coverslips (4% serum-containing medium). Arrows in siCNS point to ventral stress fibers (actin) and elongated mature FA (vinculin). White arrowheads in siILK point to a cell with an orthogonal type of actin fiber network (actin) associated with FA (vinculin), while red arrowheads point to a cell with a cortical actin ring (actin) and associated FX (vinculin). **d** IF microscopy images of ILK staining detected in siCNS and siILK cells grown for 24 h on serum-pretreated glass coverslips. Scale bar in (b, c, and d): 15 μm

**Fig. 2:**
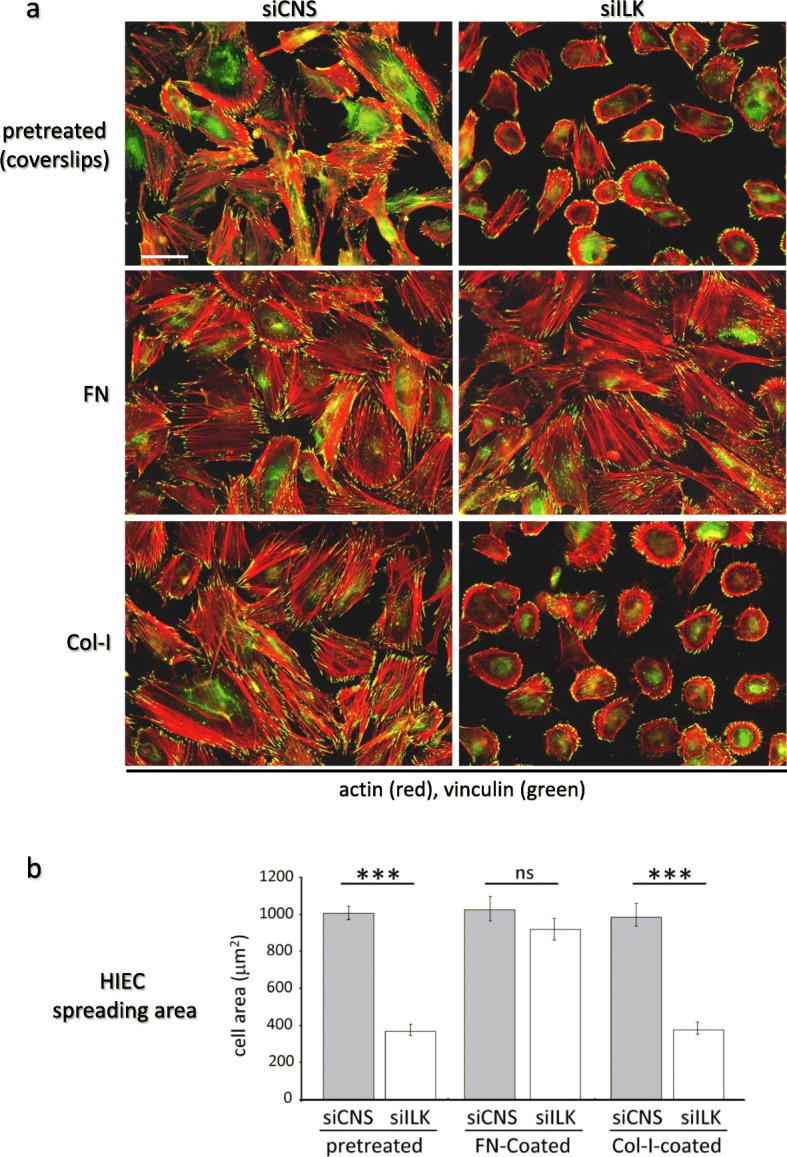
Adhesion to exogenous FN coating supports the restoration of the contractile phenotype in ILK-depleted HIEC. **a** IF microscopy images representative of the actin cytoskeleton (detected with TRITC-phalloidin; in red) and vinculin-positive ECM adhesion structures (anti-vinculin antibody; green) stained in siCNS and siILK cells grown for 24 h on serum-pretreated, FN-coated (FN; 3 μg/cm^2^) or Col-I-coated (Col-I;10 μg/cm^2^) glass coverslips. **b** Histograms of the average surface spreading area (μm^2^) measured numerically (Metamorph Imaging System software) on cells cultured under the same conditions as in (a). Results are expressed as the mean ± SEM (*n* = 3; > 50 cells have been analyzed for each condition). *** *p* < 0.001 in a one-way ANOVA using Tukey’s correction for multiple group comparisons. Scale bar in (a) 25 μm

As done previously [26], an siRNA-based approach was used to investigate the effect of ILK silencing in HIEC cells. Western blot (WB) and immunofluorescence (IF) staining confirmed that like parental HIEC cells [26], subconfluent cells transfected with a control, non-interfering siRNA abundantly expressed ILK (Fig. 1a, siCNS), which was detected diffusely at the perinuclear and cytoplasm level, and more notably in IAC (Fig. 1d, siCNS). siCNS cells also exhibited a fully spread morphology (Fig. 1b and d, siCNS) along with a contractile phenotype characterized by abundant ventral stress fibers (Fig. 1c, actin, merged, siCNS), which were in turn associated with vinculin-positive mature FA (Fig. 1c, vinculin, merged, siCNS). Conversely, we detected a substantial decrease of ILK expression by WB in siILK transfected cells (Fig. 1a, siILK), with 83.4% ± 5.8% of siILK cells displaying no staining or only faint residual IF staining of ILK (*n* = 3; 1013 cells counted in total; Fig. 1d, siILK). Additionally, the overwhelming majority of subconfluent siILK cells presented a rounded and poorly-spread morphology (Fig. 1b and 2a pretreated, siILK), along with a concordant cortical actin belt-like and/or orthogonal (transverse arcs/dorsal stress fibers) lamellipodia-type stress fiber network (Fig. 1c, actin, merged, siILK) associated with vinculin-positive FX and FA (Fig. 1c, vinculin, merged, siILK). Based on these observations, we conclude that organization in the integrin-actin axis is altered by the depletion of ILK, causing a more rounded cellular morphology in siILK cells, even though some of these cells still assemble a few ventral stress fibers. Altogether, these results point to a contribution of ILK in the acquisition (and/or maintenance) of a fully spread contractile phenotype in HIEC cells.

### Exogenous FN restores spreading capacity and associated contractile phenotype in ILK-depleted HIEC

We previously reported that ILK depletion impairs FN deposition in HIEC cells [26], consequently resulting in concomitant alterations in cell proliferation and migration capacities. Since exogenous FN rescued these aforementioned defects following ILK silencing [26], we tested whether the alterations in spreading and actin organization observed above would be likewise rescued in the same manner.

Whether grown on serum-pretreated glass coverslips (pretreated with 4% serum-containing culture medium), FN-coated or Col-I-coated glass coverslips, subconfluent siCNS cells exhibited normal spreading (as assessed by measurement of cell surface/planar areas), in addition to displaying expected ventral stress fibers associated with mature FA following IF staining (Fig. 2a, siCNS; Fig. 2b, grey columns). By comparison, an overwhelming majority of subconfluent siILK cells exhibited normal spreading and a contractile phenotype when grown on FN-coated, but not serum-pretreated or Col-I-coated glass coverslips (Fig. 2a, siILK; Fig. 2b, white columns). Hence, these results indicate that adhesion to exogenous FN rescues the spreading capacity and concomitant contractile phenotype of ILK-depleted HIEC cells.

### Deficiencies caused by ILK silencing are associated with alterations in RhoA pathway activity in HIEC cells

**Fig. 3:**
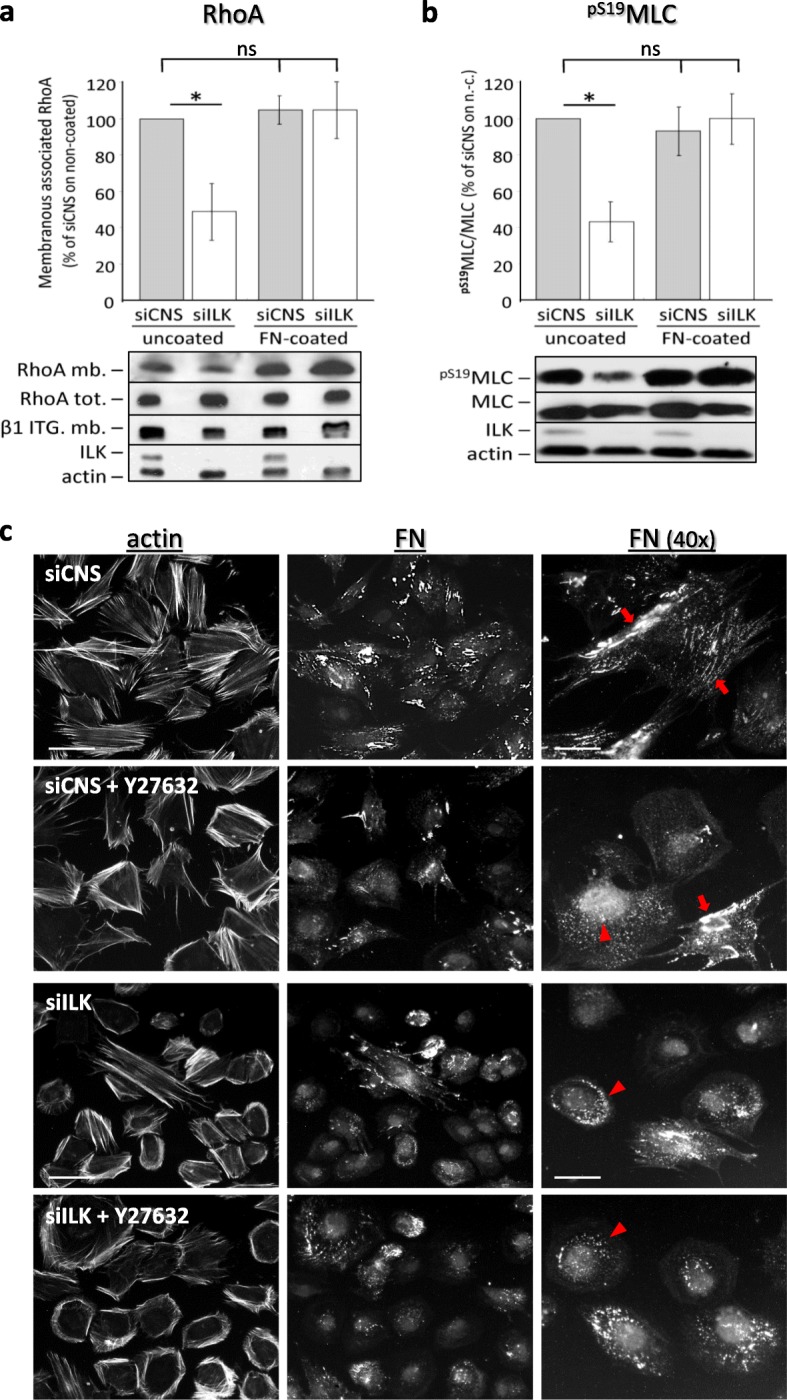
ILK silencing hinders normal FN-dependant RhoA/ROCK/MLC pathway stimulation and FN fibrillar assembly in HIEC. (a and b) siCNS and siILK cells were grown for 48 h on uncoated or FN-coated (FN-coated; 3 μg/cm^2^) plastic dishes. **a** Histograms represent the relative levels (membranous/total) of membrane-associated active RhoA (mb) compared to RhoA and β-actin detected in whole-cell lysates (tot). Values obtained by densitometry quantification analysis of WB bands were normalized to β1 integrin (from mb fractions) and β-actin (from cell lysate). The lower panels show representative corresponding protein immunoblots. **b** Histograms of the relative phosphorylation status of serine19 of the myosin light chain (^pS19^MLC/MLC) in cells cultured under the same conditions as described in (a). The values were obtained from densitometry quantification of ^pS19^MLC and MLC bands detected by WB in whole cell lysates. β-actin protein levels were used as the loading control. (a and b) Results are expressed as the percentage (%) of the values obtained for the siCNS cells on uncoated dishes ± SEM (*n* ≥ 3). * *p* < 0.05, in a one-way ANOVA using Tukey’s correction for multiple group comparisons. ns: not significant. The lower panel in (a) and (b) shows representative corresponding protein immunoblots. **c** IF microscopy images representative of the actin cytoskeleton (TRITC-phalloidin) and human FN (detected with the HFN 7.1 antibody) staining in siCNS and siILK cells grown for 24 h on serum-pretreated coverslips with or without 20 uM of the specific inhibitor Y-27632 to inhibit ROCK-mediated cell contractility. The right panels show magnification (40x objective) of FN staining, red arrows point to examples of cell-assembled FN fibrils, while the red arrowheads point to examples of non-fibrillar FN aggregates. Scale bar in (c) left panels: 20 μm; right panels: 10 μm

Since the depletion of ILK and consequent impairment of FN deposition in HIEC cells [26] led to a switch from a contractile phenotype to mostly cortical actin and/or orthogonal-type one, we investigated the status of RhoA/ROCK/MLC signaling [32, 33]. Under uncoated plastic culture conditions, siILK cells showed a significant reduction of membrane-associated/activated RhoA [34] relative levels when compared to their siCNS counterparts (Fig. 3a, uncoated). Interestingly, the RhoA decrease in siILK cells was reversed under FN-coated plastic culture conditions (Fig. 3a, FN-coated), whereas nothing changed in the case of siCNS (Fig. 3a, FN-coated). Parallel assessment of the relative levels of activated ^pS19^MLC corroborated our analyses of activated RhoA levels (Fig. 3b).

We next compared the effects of the ROCK1/ROCK2 inhibitor Y-27632 [33, 35] in subconfluent siCNS and siILK cells, grown on pretreated (but non-coated) glass coverslips. In siCNS cells, the inhibition of ROCK1/ROCK2 caused a deficiency in the formation of ventral stress fibers, although lateral/peripheral ones remained unaffected (Fig. 3c, actin, siCNS vs siCNS + Y-27632). By comparison, in siILK cells, Y-27632 did not noticeably affect their displayed deficiencies in reduced spreading and loss of ventral stress fibers, or their cortical/orthogonal-type actin organization (Fig. 3c, actin, siILK vs siILK + Y-27632). To verify any linkage with defects in FN assembly, we stained concomitantly for the visualization of deposited and cell-associated FN. In siCNS cells, the inhibition of ROCK1/ROCK2 caused a marked reduction in fibrillar FN staining (Fig. 3c, FN, siCNS vs siCNS + Y-27632), although small non-fibrillar FN aggregates were evident (Fig. 3c, FN (40x), siCNS vs siCNS + Y-27632). The loss of fibrillar FN and formation of FN aggregates were likewise observed in siILK cells, both with or without ROCK1/ROCK2 inhibition (Fig. 3c, FN, FN (40x), siILK vs siILK + Y-27632). Such aggregates partly colocalize with stress fibers in siCNS and siILK cells and likely contain partially unfolded intermediate conformation of pre-fibrillar FN [8, 12, 36], because the specific FN antibody (HFN 7.1) used herein recognizes a cryptic epitope between the PHSRN/synergy and the RGD integrin interaction domains of FN [37]. The effect of the inhibition of ROCK1/ROCK2 reveals that the RhoA/ROCK pathway is not required for FN aggregation, but is nevertheless essential for the promotion of FN fibrillogenesis in HIECs since the same inhibition markedly reduced fibrillar FN assembly in the siCNS cells.

Altogether, these results show that reduced spreading and loss of a contractile phenotype in ILK-depleted HIEC cells is associated with a deficiency in generating sufficient RhoA/ROCK/MLC pathway-mediated contributions to the development of ventral stress fibers and in FN fibrillar assembly. However, this same pathway does not appear to be required for the organization of lateral/peripheral stress fibers, for the organization of cortical/orthogonal actin, or in the aggregation of partially unfolded/pre-fibrillar FN.

### Fibrillar FN promotes spreading and a contractile phenotype independently of ILK in HIEC cells

**Fig. 4:**
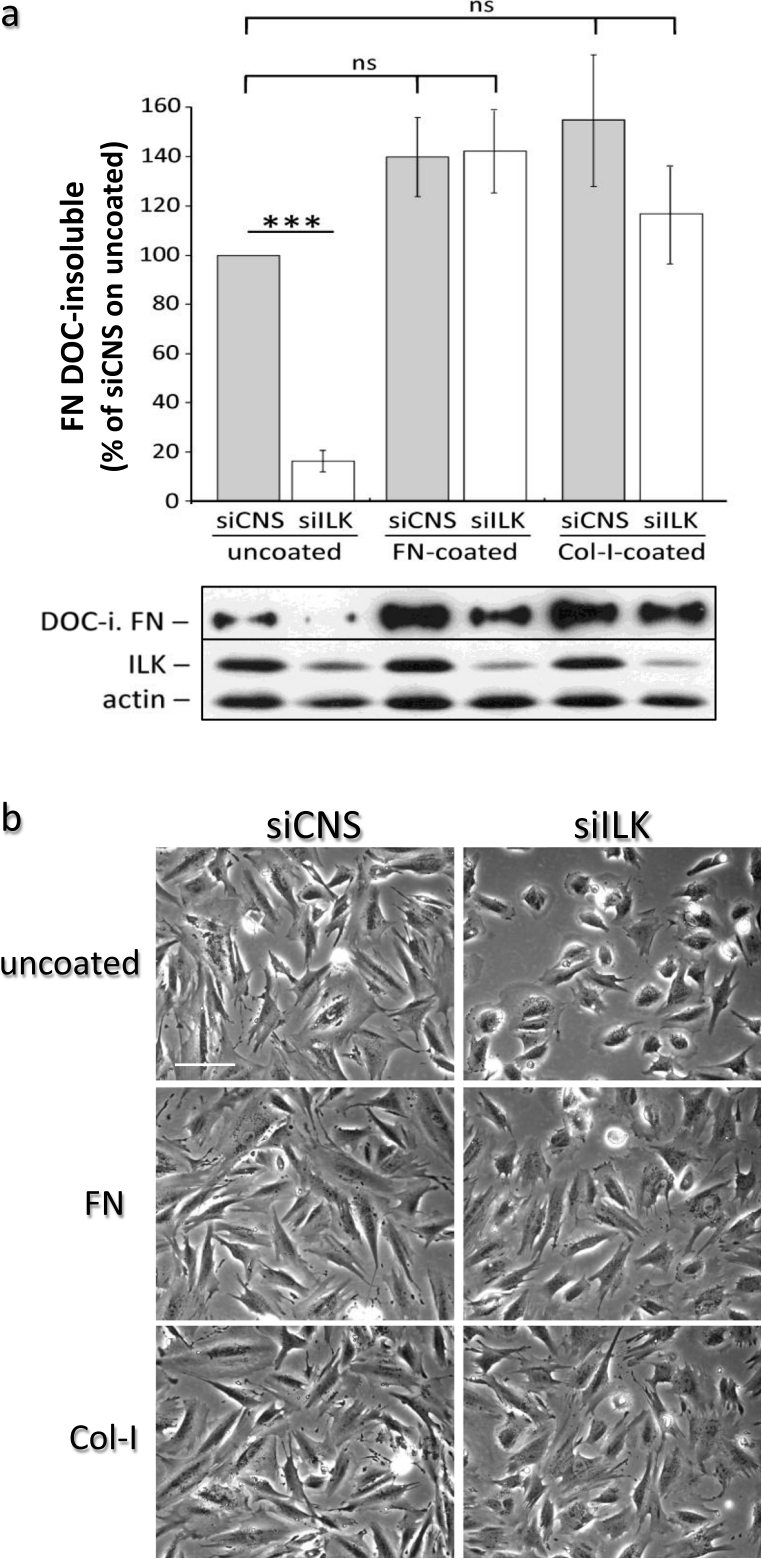
Adhesion to exogenous FN/ECM-coated substrates restores DOC-insoluble FN assembly capacity in ILK-depleted HIEC. siCNS and siILK cells were grown for 36 to 48 h on uncoated, FN-coated (3 μg/cm^2^) or Col-I-coated (10 μg/cm^2^) plastic dishes and then harvested for DOC-insoluble proteins. The histograms present the levels of isolated human DOC-insoluble FN quantified from WB densitometry analysis for each condition. The β-actin levels detected in the corresponding soluble fractions were used to normalize the DOC-insoluble levels. Results are expressed as the percentage (%) of the siCNS cells on uncoated dishes ± SEM (*n* ≥ 9). *** *p* < 0.001 in a one-way ANOVA test using Tukey’s correction for multiple group comparisons. ns: not significant. The lower panel shows representative protein immunoblots of human DOC-insoluble (DOC-i.) FN, as well as corresponding ILK and β-actin, detected from the DOC-soluble fractions. **b** Inverted contrast microscopy images representative of the same cells grown for 24 h on the same uncoated and coated plastic dish culture conditions. Scale bar in (b) 30 μm

A cell’s ability to assemble endogenous and exogenous FN seems to vary largely between cell models and culture conditions (e.g. properties of the substrate, FN: adsorbed vs in the medium) [38–42]. In order to verify whether the results described above were associated with the capacity of cells to execute FN fibrillogenesis, relative deoxycholate (DOC)-insoluble/fibrillar FN levels [43] were evaluated in siCNS and siILK cells grown on uncoated, FN-coated or Col-I-coated plastic culture dishes. As shown in Fig. 4a, a strong decrease of fibrillar FN was observed in siILK cells under uncoated conditions, as compared to their siCNS counterparts, thus confirming a link between the depletion of ILK and the loss of FN fibrillogenesis capacity. On the other hand, the siILK cells adhesion to FN- or Col-I-coated dishes led to the full restoration of DOC-insoluble/fibrillar FN levels (Fig. 4a), as well as a return to normal spreading and cell morphology in both conditions (Fig. 4b). Interestingly, the result on Col-I suggests strongly that at least in this condition, the ILK-depleted cells used their own endogenously-expressed FN to restore the fibrillogenesis process. It must be noted similar results were observed when using FN-coated glass coverslips, but not Col-I-coated ones (e.g. Fig. 2).

**Fig. 5:**
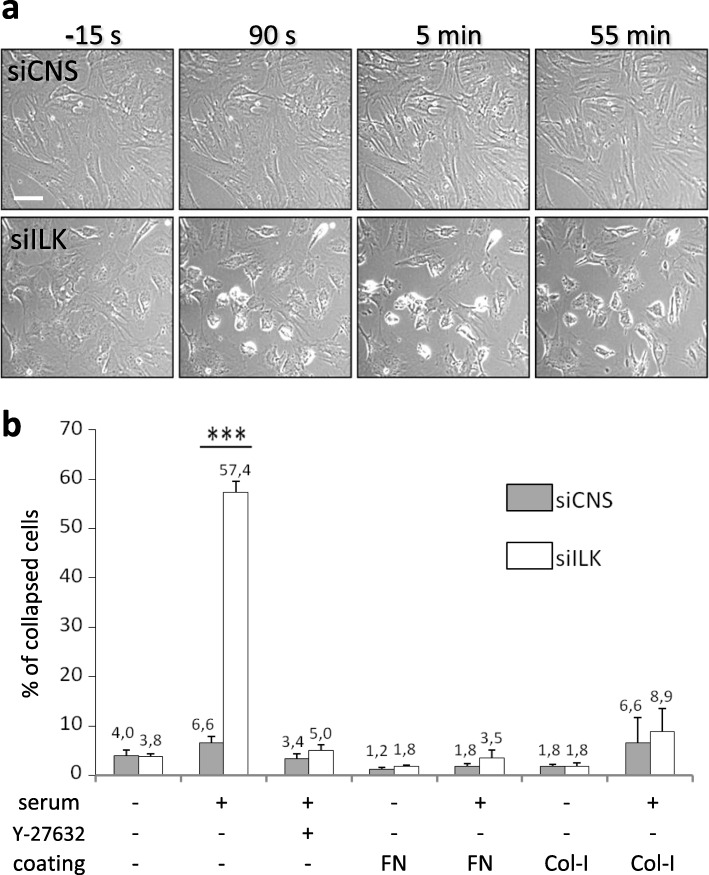
Adhesion to exogenous FN/ECM-coated substrates prevents peripheral membrane detachment and retraction following serum-induced cell contractility. siCNS and siILK cells were grown for 24 h on uncoated (a and b), FN-coated (b) or Col-I-coated (b) plastic dishes. Cells were then starved for 24 h without serum before RhoA-mediated cell contractility was stimulated by adding serum (FBS, 10% final concentration) to the culture medium. **a** Time-lapse inverted contrast microscopy images of the siCNS and siILK cells on uncoated dishes taken at a time interval of 15 s showing cells immediately before (− 15 s) and after (90 s, 5 min, and 55 min) adding the serum to the starved cells. **b** Histograms of the percentage of cells (collapsed vs total cells) that show detachment and retraction of their peripheral plasma membrane before (serum: -) and 20 to 30 min after adding the serum (+). Assays on uncoated dishes were also performed with cells pretreated with 20 μM of the ROCK inhibitor Y-27632. Results are expressed as mean ± SEM. *** *p* < 0.001 in paired t-test on the average values (siCNS vs siILK; only the statistically significant difference is indicated; *n* ≥ 3, 363 ± 151 cells counted for every specific condition in each experiment). s: second; min: minute. Scale bar in (a): 20 μm

We next evaluated the capacity of starved siCNS and siILK cells to maintain their adhesion following the stimulation of RhoA-mediated contractility by serum [44], whether these cells were previously grown on uncoated, FN-coated or Col-I-coated plastic dishes. Several soluble components of serum (e.g. lysophosphatidic acid) can induce specific cell membrane receptors (e.g. G protein-coupled receptors) leading to a rapid increase of RhoA/ROCK-dependant actomyosin contractility and isometric tension applied on IAC by stress fibers [44–47]. Under uncoated conditions and following serum stimulation, time-lapse microscopy revealed a majority of siILK cells displayed a rapid peripheral IAC detachment and membrane retraction-like/collapse phenomenon at 90 s, which peaked around 5 min and persisted until the affected cells gradually began to spread anew, as observed 55 min after the initial serum stimulation (Fig. 5a, siILK; Fig. 5b). By contrast, only limited membrane retraction between peripheral IAC was observed in siCNS cells following serum stimulation (Fig. 5a, siCNS; Fig. 5b) and exposure to Y-27632, like adhesion to FN-coated or Col-I-coated plastic dishes, prevented the retraction-like/collapse phenomenon caused by the serum in siILK cells (Fig. 5b). Since FN fibrillogenesis is only restored in siILK cells that were grown under coated conditions (see Fig. 4a), these results suggest that the defects in FN fibrillogenesis caused by ILK-depletion greatly hinder the mechanotensile anchoring resilience of the siILK cells grown on uncoated dishes, therefore causing peripheral IAC detachment from the ECM when a rapid increase in cell contractility is forced by the stimulation with serum. Altogether, these results show that fibrillar FN promotes cell spreading and a contractile phenotype independently of ILK, in HIEC cells.

### ILK induces the fibrillogenesis of endogenous FN, but the fibrillogenesis process is ILK-independent in HIEC cells

**Fig. 6:**
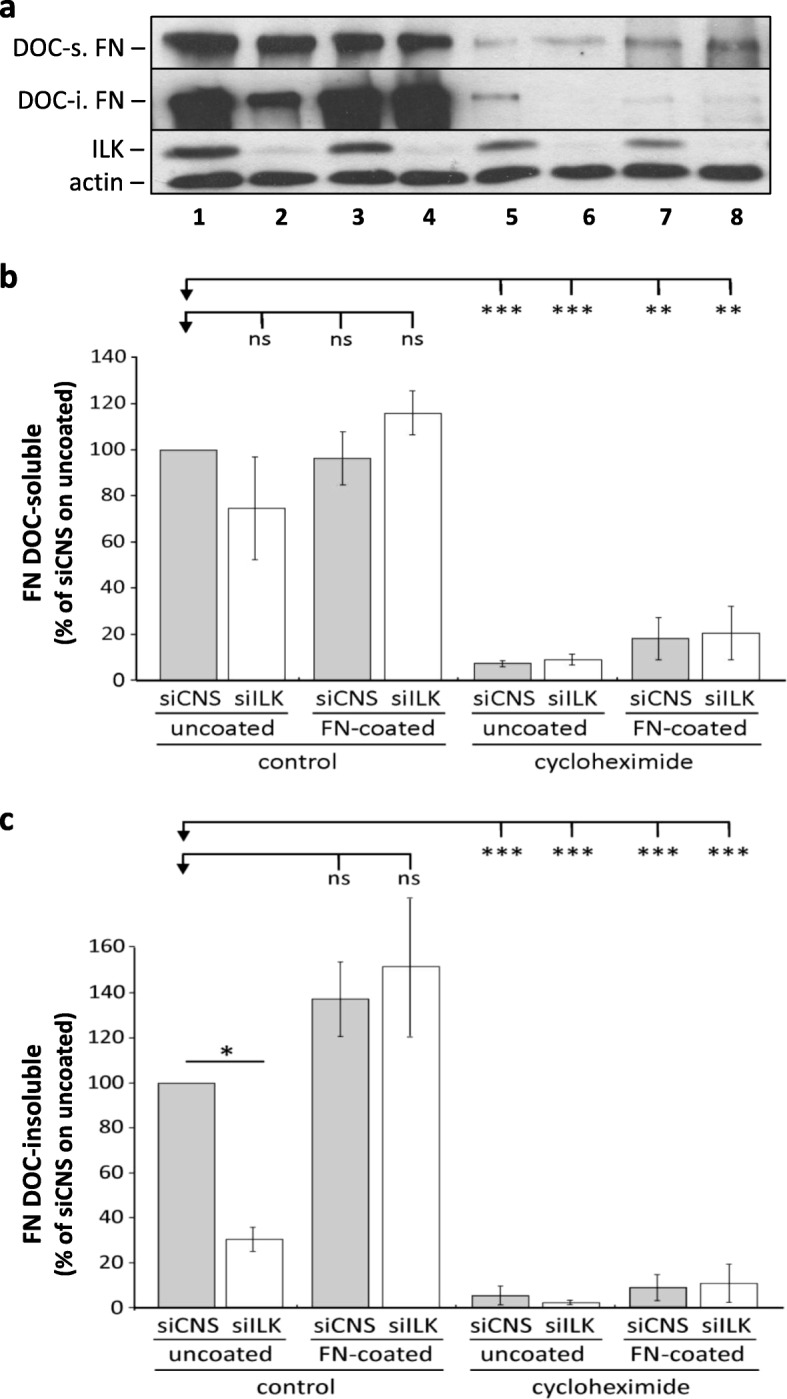
Inhibition of FN synthesis with cycloheximide prevents FN fibrillogenesis in ILK-depleted HIEC on exogenous FN. (a, b and c) siCNS and siILK cells were grown for 24 h on uncoated or FN-coated plastic dishes, with or without cycloheximide (10 μg/ml) and were harvested for DOC-insoluble proteins. **a** Representative WB of human FN (HFN 7.1 antibody) detected in DOC-soluble (DOC-s.) and DOC-insoluble (DOC-i.) fractions, as well as the corresponding ILK and β-actin protein immunoblots detected from the DOC-s. fractions. The order of WB bands in (a) follows the order of the histograms presented in (b and c). (b and c) Histograms of the levels of human FN quantified from WB densitometry analysis of siCNS and siILK cells **b** DOC-soluble and **c** DOC-insoluble fractions. β-actin detected in the soluble fractions was used to normalize the results for each experiment. Results are expressed as the percentage (%) of the siCNS cells on uncoated dishes ± SEM. (*n* = 4). *** *p* < 0.001, ** *p* < 0.01 and * *p* < 0.05 in a one-way ANOVA test using Newman-Keuls correction for multiple group comparisons. ns: not significant

We subsequently analyzed the impact of ILK depletion on the relative levels of DOC-soluble and DOC-insoluble FN following the inhibition of endogenous FN synthesis with cycloheximide [35], under uncoated or FN-coated plastic culture conditions. We found there was no significant difference between the levels of soluble FN detected in siCNS and siILK cells, as well as between uncoated and FN-coated conditions, whether FN synthesis was blocked or not (Fig. 6a-b). In contrast, and as expected from the results shown above, significant differences in relative levels of insoluble/fibrillar FN were noted between siCNS and siILK cells under uncoated conditions while no difference was seen on FN-coated ones (Fig. 6a, c). Furthermore, the inhibition of FN synthesis resulted in an important reduction of both soluble and insoluble FN levels in siCNS and siILK cells, regardless of whether they were grown under uncoated or FN-coated conditions (Fig. 6a-c). These results confirm that HIEC cells use their endogenous soluble FN for assembly into a fibrillar matrix, independently of ILK expression. However, in the absence of exogenous FN/ECM coatings, ILK is required to induce this process.

**Fig. 7:**
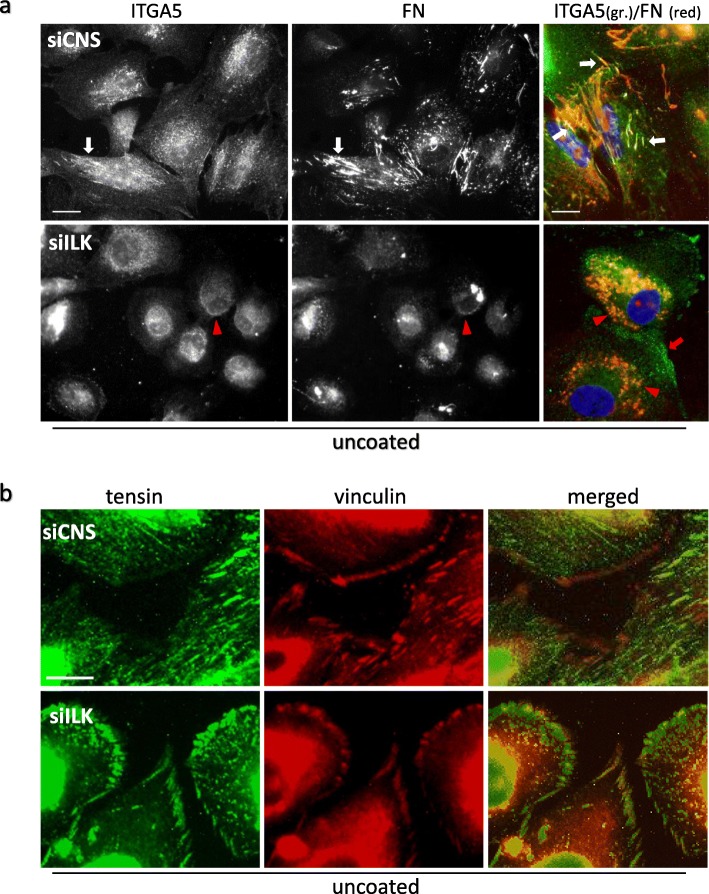
Effect of ILK silencing on α5β1 integrin, FN, and tensin distribution in HIEC. (a and b) siCNS and siILK cells were grown for 24 h on serum-pretreated glass coverslips and then fixed and prepared for IF staining. **a** Co-IF microscopy images representative of α5β1 integrin (ITGA5; α5 subunit detected with the AB1928 antibody) and cell-associated human FN (HFN 7.1 antibody) staining distribution in siCNS and siILK cells. The right panels show merged and magnified co-detection (ITGA5 in green, FN red, DAPI staining of the nucleus in blue). White arrows in siCNS points to ITGA5 and FN colocalization in fibrillar structures. Red arrows and red arrowheads in siILK point respectively to subcortical staining of α5 integrin and its colocalization with small punctuated aggregates of FN. **b** Co-IF microscopy images representative of tensin (green) and vinculin (red) staining distribution in siCNS and siILK cells. Scale bar in (a): 10 μm; (b): 5 μm

**Fig. 8:**
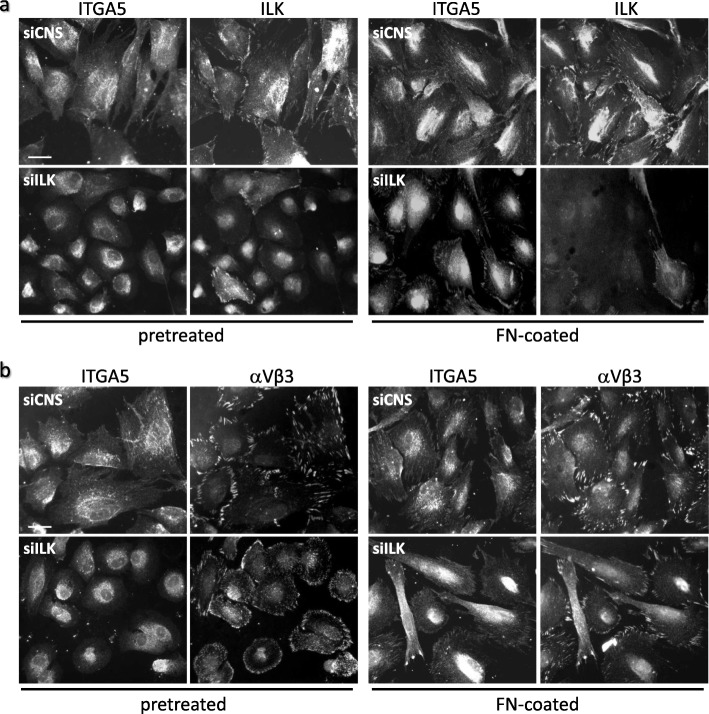
Adhesion to exogenous FN restores normal α5β1 and αVβ3 integrin distribution in ILK-depleted HIEC. siCNS and siILK cells were grown for 24 h on serum-pretreated or FN-coated glass coverslips and then fixed and prepared for IF staining. (a and b) Co-IF microscopy images representative of α5β1 integrin (ITGA5; α5 subunit/AB1928 antibody) staining with **a** ILK staining, and **b** αVβ3 staining distribution in siCNS and siILK cells. Scale bar in (a and b): 15 μm

Since FN fibrillogenesis is ILK-independent, the question remained as to the apparent functional association between ILK depletion in cells and their loss of capacity to deposit [26] and assemble soluble FN into a fibrillar matrix (this study). Co-IF staining in siCNS cells grown on serum-pretreated glass coverslips confirmed that the α5 subunit of the integrin α5β1 colocalized with assembled FN (Fig. 7a, siCNS), and with ILK in fibrillar structures (Fig. 8a, pretreated, siCNS). As expected [33], tensin staining was likewise found principally in comparable fibrillar structures, out of the vinculin-positive FA (Fig. 7b, siCNS), while on the contrary, αV of the αV-class integrins (e.g. αVβ1/β3/β5) was detected at large peripheral FA (Fig. 8b, pretreated, siCNS). In contrast, siILK cells grown under the same conditions showed a redistribution of α5β1 at punctuated sites comparable to previously observed unfolded/pre-fibrillar FN aggregates (Fig. 7a, siILK). The bulk of tensin staining was redistributed at vinculin-positive FA (Fig. 7b, siILK). Although most αV detection remained unchanged, some of its staining was also redistributed to sparse small aggregates (Fig. 8b, pretreated, siILK). We next compared pretreated and FN-coated glass coverslip culture conditions. Except for the emergence of a weak signal for α5β1 in peripheral IAC, siCNS cells showed no other notable changes in staining for α5β1, ILK, and αV (Fig. 8a-b, FN-coated, siCNS). In the case of siILK cells exposed to exogenous FN, the stainings for α5β1 and αV (Fig. 8a-b, FN-coated, siILK), as well as tensin (see Additional file 1), were found to be similar to those noted for their siCNS counterparts, thus indicating the return to the normal localization of these proteins under this culture condition.

**Fig. 9:**
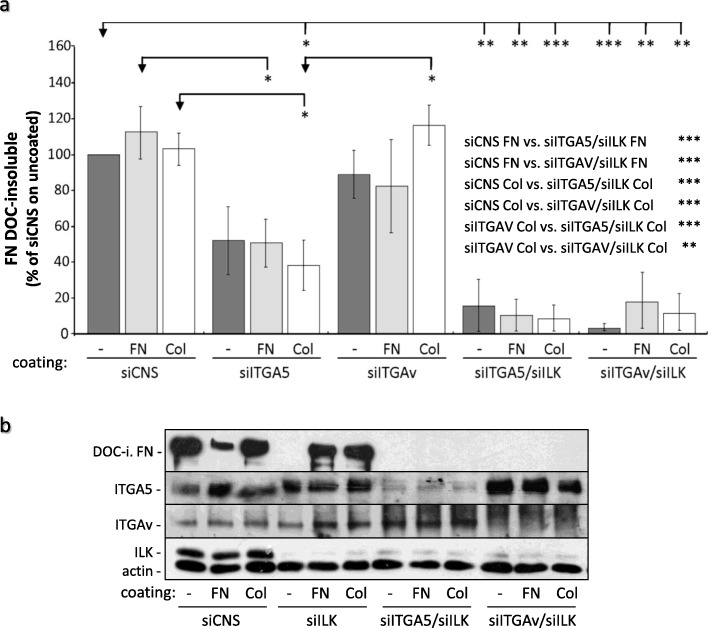
Effect of α5 or αV integrins and concomitant ILK silencing on FN assembly in HIEC. (a and b) HIEC cells were transfected with 20 nM of siCNS, siILK or siRNA directed against the expression of α5 (siITGA5) and αV (siITGAV) integrin subunits alone or in combination with 20 nM of siILK (e.g. siITGA5/siILK). The cells were subsequently plated and grown for 48 h on uncoated, FN-coated and Col-I coated plastic dishes before being harvested for DOC-insoluble protein extraction. **a** The histograms present the levels of human DOC-insoluble FN quantified from densitometry analysis of WB bands. β-actin detected in the soluble fractions was used to normalize the results for each experiment. Results are expressed as the percentage (%) of the siCNS on uncoated dishes ± SEM (*n* ≥ 3). * *p* < 0.05, ** *p* < 0.01 and *** *p* < 0.001 in a one-way ANOVA test using Tukey’s correction for multiple group comparisons (only the statistically significant differences are indicated). **b** Representative protein immunoblots of DOC-insoluble (DOC-i.) human FN and the corresponding ILK, β-actin, α5 (ITGA5) and αV (ITGAV) integrin subunits detected from the DOC-soluble fractions of siCNS, siILK, siITGA5/siILK and siITGAV/siILK cells

We subsequently analyzed the relative levels of fibrillar FN assembled by HIEC cells following siRNA-mediated silencing of their expression of the α5 (siITGA5) or αV (siITGAV) integrin subunits. As reported in other cell types [48], some compensatory upregulation of αV integrin expression was observed in siITGA5 cells and vice-versa. In any case, the depletion of ITGA5 caused a significant reduction in DOC-insoluble FN levels regardless of whether cells were grown on uncoated, FN-coated or Col-I-coated plastic culture dishes (Fig. 9a; siCNS vs siITGA5). In contrast, no significant reduction of insoluble/fibrillar FN levels was noted in siITGAV cells (Fig. 9a; siCNS vs siITGAV). However, the concomitant depletion of ILK with that of either α5 or αV integrin subunits prevented the restoration of DOC-insoluble fibrillar FN levels by exogenous FN or Col-I coatings (Fig. 9a-b). It is germane to specify that no apparent increase in cell detachment/death (e.g. numerous floating cells) was observed following the silencing of either ILK, ITGA5 and/or ITGAV. Arguably, our assays were performed in the presence of serum which helps the survival of intestinal cells in culture [49, 50], and intestinal epithelial crypt cells express several other ECM proteins (e.g. laminins, collagens) and integrins (e.g. α2β1, α6β4) that can support cell adhesion and also signal to prevent anoikis [50–52]. However, since we did not specifically monitor the rate of apoptosis/anoikis levels, we cannot exclude that the depletion of these proteins had any impact on cell survival.

These findings indicate that FN fibrillogenesis is executed mainly via the α5β1 integrin, although α5β1 and αV-class integrins may compensate for each other at least partially, in addition to compensating fully for the loss of ILK in the execution of the same process within the context of cell adhesion to exogenous FN/ECM. Furthermore, these results taken together with the rest of the findings presented herein show that ILK expression induces the fibrillar assembly of endogenous soluble FN which, in turn, and independently of ILK, promotes maturation of IAC and stress fibers, and the acquisition of a fully spread contractile phenotype.

## Discussion

In the present study, we show that ILK supports the contractile phenotype in HIEC cells primarily by the downstream effects of its contribution to the induction of FN fibrillogenesis. Studies have revealed a close functional relationship between integrin adhesion, RhoA signaling, fibrillar FN assembly, and the contractile phenotype in cells [13, 17, 33, 35, 53, 54]. ILK, through the IPP complex it forms within adhesomes, is a major participant in integrin-mediated cellular function and processes, including actin organization and contractility [23, 24]. However, the mechanisms by which ILK can regulate RhoA/ROCK pathway signaling and FN fibrillogenesis have remained unclear [23].

Studies have shown that FN fibrillogenesis correlates with increased ECM stiffness [54], while ECM properties (e.g. stiffness, ligand spacing) regulate IAC-mediated signaling that determines force loading by stress fibers and final steady-state cellular morphology [3, 4, 55]. Indeed, ILK-depleted crypt cells grown on exogenous FN not only display restored FN fibrillogenesis but also a concomitant rescue of the RhoA/ROCK/MLC pathway, spreading and the contractile phenotype (i.e. with ventral stress fibers) alongside the restoration of cell mechanotensile resilience (as observed by the prevention of IAC detachment and membrane retraction following serum-stimulation). Altogether this confirms the reinstated generation of strong and functionally-competent ECM-integrin-actin axes, which in turn reveals that in HIEC cells ILK is only required indirectly for the optimal engagement of the RhoA/ROCK/MLC pathway and seemingly not for its putative role in the stabilization of integrin-actin linkage [23]. However, since ILK is still faintly detected in the FA of the ILK-silenced HIEC we cannot completely exclude the possibility that this remaining pool contributes to mediate the downstream effect of cell adhesion to the FN matrix.

Whereas some fibroblastic models have shown the capacity to assemble fibrils directly from the underlying adsorbed FN or from soluble FN added to the medium [38–42], it has been reported that bovine aortic endothelial (BAE) cells are unable to assemble exogenous adsorbed FN nor soluble FN added to the medium and solely rely on endogenous FN secreted basally to assemble a fibrillar matrix [56]. Similarly, we observed that like endogenous soluble FN secreted in the medium of ILK-depleted HIECs [26], exogenous serum plasma FN supplied directly to the medium of these cells does not help rescue the contractile phenotype (see Additional file 2) thus indicating that ILK-depletion hinders HIEC ability to use both endogenous and exogenous soluble FN. Additionally, our observations that inhibition of FN synthesis by cycloheximide in HIEC cells resulted in the absence of FN fibrillogenesis, regardless of whether ILK was depleted or whether the cells were grown on exogenous FN (or Col-I), reveal that: 1) little if any exogenous FN from the adsorbed coating was assembled in the DOC-insoluble matrix, and therefore 2) these cells used preferentially their endogenously-expressed FN to form their fibrillar ECM. Incidentally, and as is the case for exogenous FN coated on either plastic or glass surfaces, we found that such rescue of fibrillar FN assembly by Col-I is contingent upon endogenously produced soluble FN, whether the cells are ILK-depleted or not. It is reasonable to assume that cell-type specific mechanisms regarding the cellular contribution of ILK exist since numerous integrin/actin-related defects (e.g. formation of FBs, FN fibrillogenesis, spreading, etc.) caused by functional inactivation/depletion of ILK in other cell types/species have been reported by several studies using cells on FN-coated substrate [23, 57–60]. Studies with mouse embryonic fibroblasts (MEF) [40, 57] and BAE cells [56, 58] found a very similar impact between ILK and FN expression inhibition on the respective phenotype of these models, leading to the suggestion that the role of ILK in FN assembly can explain at least some of these similarities [40, 56].

In addition to α5β1, HIEC cells express a subset of αV- and β1- class integrins which can be used for binding to RGD motif-containing ECM components and/or FN-dependent adhesion [32, 35]. As expected [33], we confirm α5β1 as the main integrin used for FN fibrillogenesis in HIEC cells, and likewise, its co-distribution with ILK and FN in more central fibrillar structures. While ILK is also detected at peripheral IAC, as described by Pankov et al. (2000) for α5β1 [7], these observations suggest that some ILK could translocate with α5β1 out of the FA or at least be recruited to this specific integrin population during FN assembly and consequently may be involved in FB formation as reported in other cell models [56, 59, 60]. However, the fact that exogenous FN, or Col-I, restore the establishment of ventral stress fibers and tensin-positive fibrillar structures in ILK-silenced cells leads to the assumption that ILK is not directly required for the formation of FB in HIEC.

Although the expression of the α5 and αV integrin subunits was unaffected following ILK depletion [26], the structural (e.g. cortical/orthogonal actin fibers network) and morphological (rounded) characteristics of the ILK-depleted cells were representative of immature cells spreading [6, 61] and predominant adhesion by the αV-class integrins (e.g. αVβ3) [13, 62, 63]. The altered distribution of the integrins staining in these cells was seemingly related to the inhibition of fibrillogenesis, still, we denoted that the α5β1 punctuated staining partly co-localizes with FN aggregates of partially unfolded/pre-fibrillar FN – as indicated through our use of the HFN 7.1 antibody [37]. Notably, when colocalized they frequently aligned with the integrin-actin structures in areas where cell-associated FN is reportedly initially stretched before fibril assembly [36], indicating that the depletion of ILK does not fully prevent early interactions between FN and α5β1, nor α5β1 linkage to the actin cytoskeleton. These results are consistent with a model in which ILK-depletion hampers specifically α5β1-mediated FN assembly at an early stage of the fibrillogenesis process. This, however, do not preclude that synergistic crosstalk between αV-class integrins and developing α5β1-FN adhesion sites [15, 20–22] could promote ILK-induced fibrillogenesis since the concomitant depletion of ILK with the αV-class integrins, like with the α5 integrin, almost completely abrogates the assembly of DOC-insoluble FN regardless of the culture conditions. Conversely, the depletion of αV alone did not have any impact on the DOC-insoluble levels, suggesting that αV-class integrins functions might nevertheless be dispensable in HIEC cells, especially considering that these cells express abundantly the integrin α8β1, another RGD-dependent receptor that mediates FN adhesion and RhoA signaling [32, 35].

ILK through the IPP complex may regulate integrin function directly [64, 65] or indirectly by recruiting other important adhesome components (e.g. Kindlin-2) [66–68], as well as directing the molecular packaging of IAC [60] and the assembly of specific F-actin bundles in stress fibers [25]. This raises the question of the specific functional contribution of ILK to fibrillogenesis in HIECs. We noted that the rescue of this process by exogenous Col-I occurred only with plastic dishes. It is well known that exogenous proteins adsorb differently on surfaces with distinct properties which can cause variations in the properties (e.g. density, rigidity, topography, conformation and interactions) of the protein forming the coating, which in turn direct cell activity [69–72], including FN deposition and fibrillogenesis [73–76]. For example, the conformational change induced by the adsorption of soluble FN to plastic or its specific interaction with Col-I adsorbed to plastic prompt the binding and activation of α5β1 [[37, 77, 78] and cell contractility [79]. Also, Col-I adsorption on plastic is reported to saturate at a much higher density compared to glass [72]. The properties (e.g. ligand density, capacity to induce functional FN conformation) of the FN-coating and Col-I-coated plastic (but not Col-I-coated glass) may help to restore the accessibility or availability of specific FN interaction sites and therefore explain the ability of these substrates to bypass the role of ILK in fibrillogenesis and engage the contractile machinery [60, 80, 81]. This supports a model in which, during the early phases of HIEC spreading, ILK is recruited to newly form IAC direct integrin capacity to optimally bind to endogenous FN (e.g. RGD, PHSRN/synergy) and the α5β1-FN interactions that are normally required to induce the fibrillogenesis process. This function of ILK is in all likelihood performed through the IPP complex as in a previous study we have shown that the majority of the ILK expressed by the HIEC cells is complexed to PINCH and parvin proteins, that also localize at IAC [26].

## Conclusion

The main finding of this study in HIEC cells is that ILK’s contributions to the initiation of endogenous FN fibrillogenesis support a functionally-competent ECM-integrin-actin axis, which in turn, and independently of any other potential direct ILK function, leads to the acquisition and maintenance of a fully spread contractile phenotype. The functional relationship between ILK and FN fibrillogenesis, as observed herein with HIEC, is different from those reported previously with other cell models and constitutes further justification for acquiring a better understanding of the roles of ILK in ECM-integrin-actin interaction dynamics – especially in the context of intestinal epithelial mechanobiology with regards to homeostasis and disease states. Indeed, our results suggest that ILK can be a particularly interesting target to evaluate for the treatment of intestinal pathologies such as colon cancer and inflammatory bowel diseases since deregulation of fibronectin, integrin α5β1 and RhoA function or expression have been shown to contribute to the progression of colon cancer and poor prognosis [82–84], while abnormal assembly of FN can trigger intestinal tumor invasion [85] and excessive collagen deposition associated with the formation of fibrous lesions and tissues fibrosis in inflammatory condition [86, 87].

## Methods

### Materials

Mouse primary antibodies used were directed against: ILK (WB:1/1000; clone3/ILK, BD Transduction Laboratories, Franklin Lakes, NJ), β-actin (WB:1/75000; clone C4, Santa Cruz Biotechnology Inc., Dallas, TX), human FN (WB:1/500, IF:1/100; HFN 7.1, Developmental Studies Hybridoma Bank, Iowa City, IA), αV integrin (WB:1/1000, IF:1/200; clone P3G8, MilliporeSigma), vinculin (IF:1/500; clone 7F9, from Chemicon’s FAK100 kit, MilliporeSigma, Cleveland, OH) and tensin1 (WB:1/1000, IF:1/100; a kind gift from Su Hao Lo, Ph.D, Department of Biochemistry and Molecular Medicine, University of California-Davis, Sacramento, CA), RhoA (WB:1/100; 26C4, Santa Cruz Biotechnology Inc.). Rat primary antibody used was directed against β1 integrin (WB:1/1000; mAB13, a kind gift from Kenneth Yamada, MD, Ph.D., NIH & NIDCR, Bethesda, MD). Rabbit primary antibodies used were directed against: α5 integrin (WB:1/1000, IF:1/100; AB1928, MilliporeSigma), MLC2 (WB: 1/1000; 3672, Cell Signaling Tech., Danvers, MA), and ^pS19^MLC2 (WB:1/1000; 3671, Cell Signaling Tech.). Secondary antibodies used were: goat anti-mouse and goat anti-rabbit Alexa Fluor® 488 (Invitrogen, Carlsbad, CA), goat anti-mouse Alexa Fluor® 594 (Invitrogen) and sheep anti-mouse rhodamine conjugate (MilliporeSigma). Actin IF staining was performed with TRITC-conjugated phalloidin (from Chemicon’s FAK100 kit, MilliporeSigma). Pharmacological inhibitors used were: the ROCK-1/− 2 inhibitor, Y-27632 (20 μg/ml; MilliporeSigma) [33, 35]. All other materials were purchased from MP Biomedicals, BD Biosciences, MilliporeSigma, or Thermo-Fisher Scientific (Ottawa, ON, Canada), except where otherwise specified.

### HIEC cell culture

The HIEC-6 cell line is a human intestinal epithelial crypt (HIEC) cell model. These non-transformed and non-immortalized cells express a phenotype corresponding to normal human proliferative/undifferentiated crypt cells [88, 89]. HIEC-6 cells were routinely cultured in 100 mm plastic dishes (Falcon Plastics Inc., Los Angeles, CA) with Opti-MEM® (Invitrogen) containing 4% fetal bovine serum (FBS/serum; CELLect Gold™, MP Biomedicals Inc., Santa Ana, CA) culture medium and maintained as described elsewhere [90].

### siRNA transfection

Silencer® Select validated siRNAs (highest target knockdown and specificity) against ILK (s7404), α5 integrin (s7547) and αV integrin (s7569), as well as a control non-silencing siRNA (siCNS; AM4611), were from ThermoFisher Scientific. One day prior to transfection, cells were plated at low density in 100 mm plastic dishes. As described previously [26], cells were transfected for 5 h without serum with the X-tremeGENE siRNA transfection reagent (Roche Diagnostics Canada, Laval, QC), using a final concentration of 20 nM for each specific siRNA, before replacing the transfection medium with regular culture medium. Cells were thereafter used for analyses/experiments 48 h post-transfection, as determined elsewhere [26]. The higher efficiency of the siRNAs used in this study (e.g. ILK: Silencer® Select ID s7404 vs Silencer® ID 1461 [26]; ThermoFisher Scientific) explains why we were able to lower the concentration (from 40 nM to 20 nM) of the siRNA transfected compared to what we described previously [26].

### Exogenous FN and col-I assays

12 mm diameter glass coverslips (Fisherbrand™, Thermo-Fisher Scientific) were sterilized with 70% ethanol solution and placed at the bottom of 12-well Falcon® plates (Falcon Plastics Inc., Brookings, SD). The coverslips were then pre-treated with 4% serum in Opti-MEM® (Invitrogen) or coated with 3 μg/cm^2^ of human plasma FN (MilliporeSigma) in PBS (pH 7.4), or 10 μg/cm^2^ of rat tail Col-I (BD Biosciences, Bedford, MA) in 0.02 N acetic acid/H_2_O solution, for at least 2 h at 37 °C. These were followed with 2% BSA-PBS (pH 7.4) for 1 h at 37 °C prior to cell seeding [26]. The same procedure was used for the coating of 100 mm or 60 mm plastic dishes.

### WB analyses

Cell cultures were lysed in 1x Laemmli, reduced with 5% β-mercaptoethanol and processed as previously described [91]. Total proteins (100 μg/lane) were resolved by SDS-PAGE on 12 to 15% gels, electrotransferred onto nitrocellulose membranes (Bio-Rad Laboratories, Mississauga, ON) and probed as established previously [91]. Full-range Rainbow Marker molecular mass markers (GE Healthcare Bio-Sciences, Baie-d’Urfé, QC) or Precision Plus Protein Dual Color Standards (Bio-Rad) were used as standards. Immunoreactive bands were visualized using the enhanced chemiluminescence (ECL) method according to the manufacturer’s instructions (GE Healthcare) and as described previously [89]. WB band intensities were determined by densitometry using Scion Image (Scion Corporation, Frederick, MD) software. Relative expression levels were established with the ratio protein of interest/actin; relative activated/phosphorylated levels were established with the ratio phosphorylated protein of interest/total protein of interest – as previously described [33].

### Optical microscopy and IF staining

For indirect IF, cells were cultured on pretreated or coated-glass coverslips (12 mm) and processed as previously described [35]. Both primary and secondary antibodies were diluted in PBS (pH 7.4) containing 5% BLOTTO or 2% BSA. For some experiments, nuclei were counterstained with 10 ng/ml 4′,6-diamidino-2-phenylindole (DAPI)-PBS (pH 7.4) and samples were mounted in glycerol-PBS (9:1) containing 0.1% phenylenediamine. IF signals were viewed with a DM RXA microscope (Leica, St-Laurent, QC, Canada) equipped for epifluorescence and digital imaging (MicroMax 1300YHS cooled CCD; Princeton Instruments, Trenton, NJ). Numerical images were processed using the MetaMorph Imaging System (Universal Imaging Corp., West Chester, PA) and the Adobe® Photoshop® software (Adobe, San Jose, CA). Cell spreading areas were measured numerically using the MetaMorph Imaging System (Universal Imaging Corp.). For optical inverted contrast microscopy, cells were cultured on plastic culture dishes and viewed with a DM IRBE microscope (Leica) equipped for digital imaging (Princeton Instruments).

### RhoA serum-stimulation assays

Cells were plated on uncoated or FN- or Col-I-coated plastic dishes and then cultured for 24 h before being starved without serum for an additional 24 h, prior to adding serum back to the medium (10% FBS final concentration) in order to induce RhoA-mediated cell contractility [92]. For some experiments, starved cells plated on uncoated surfaces were treated with Y-27632 30 min before the induction of cell contractility with serum. Random field inverted contrast images were taken approximately 20 to 30 min after the start of the assays to determine the number of retracted/collapsed cells over the total number of cells counted per field. Time-lapse images were taken at time intervals of 15 s prior to adding serum, immediately after adding serum (0 s), and subsequently at every 15 s henceforth to a maximum time of 55 min.

### Membrane-associated RhoA analyses

Cells were rinsed with cold PBS and incubated in lysis buffer (250 mM sucrose, 10 mM Tris pH 7.5, 1 mM PMSF and commercial protease inhibitor cocktail) before being transferred to an Eppendorf. After 3 cycles of liquid nitrogen freezing and thawing (at 37 °C), the samples were transferred and centrifuged at 100,000 x g for 1 h at 4 °C. The pellets, enriched in plasma membrane proteins, were washed with lysis buffer and resuspended in 100 μl of this buffer supplemented with 0.1% of SDS and 1% of Triton X-100. RhoA signals detected by WB in the membrane-enriched fractions and the corresponding total lysates were compared and normalized to β-actin detected in the total lysates in order to determine the relative levels of (active) membrane-associated RhoA, as described elsewhere [34].

### DOC-insoluble FN assembly analyses

Cells were plated at high density onto 60 mm uncoated or FN- or Col-I coated plastic dishes and cultured for 36 to 48 h before being harvested as described elsewhere [43]. When cycloheximide (10 μg/ml) was used in order to block endogenous FN synthesis, cells were cultured for only 24 h before being harvested [35, 43]. DOC-insoluble protein extraction was performed on the harvested cell lysate according to a method described elsewhere [43]. Relative DOC-insoluble FN levels were monitored by WB and normalized with the corresponding β-actin detected from the DOC-soluble fraction.

## Supplementary information

**Additional file 1.** ILK-depletion does not prevent tensin recruitment to peripheral adhesion sites in HIECs. (a) Western blot analysis shows comparable levels of tensin detected in siCNS and siILK cells. (b) Epifluorescence microscopy images representative of tensin distribution siCNS and siILK cells growth on pretreated and FN-coated (3 μg/cm^2^) glass coverslips. Adhesion to FN restores a fibrillar distribution of tensin in HIEC siILK cells. Scale bar in (b): 10 μm.

**Additional file 2.** Adding exogenous soluble FN to the medium does not rescue the contractile phenotype in ILK-depleted HIECs. (a) Inverted contrast microscopy images of siCNS and siILK plated and grown for 24 h on uncoated plastic dishes before (upper panels) and 24 h after (lower panels) adding medium containing human plasma FN (17 μg/ml final concentration ≅ 3 μg/cm^2^). (b) The cells were plated on uncoated (upper panels) and FN-coated (3 μg/cm^2^; lower panels) dishes 4 h before adding human plasma FN to the medium and grown for an additional 24 h under these conditions. Scale bars in (a and b): 30 μm.

## Data Availability

The data that support the findings of the current study are available from David Gagné or the corresponding author upon reasonable request.

## Abbreviations

Col-I: Collagen-I
DAPI: 4′,6-diamidino-2-phenylindole
DOC: Deoxycholate
DOC-i./−s: Deoxycholate insoluble/soluble
ECL: Enhanced chemiluminescence
ECM: Extracellular matrix
FA: Focal adhesions
F-actin: Actin filaments
FAK: Focal adhesion kinase
FB: Fibrillar adhesions
FBS: Fetal Bovine Serum
FN: Fibronectin
FX: Focal complexes
HIEC: Human intestinal epithelial crypt
IAC: Integrin adhesion complex
IF: Immunofluorescence
ILK: Integrin-linked kinase
IPP: ILK-PINCH-parvin complex
ITGA5: Integrin α5 subunit
ITGAV: Integrin αV subunit
MLC: Myosin light chain
PBS: Phosphate buffer saline
PHSRN: Pro-His-Ser-Arg-Asn
PINCH: Particularly Interesting new cys-his protein
RGD: Arg-GLY-ASP
RhoA: Ras homolog gene family member A
ROCK: Rho-associated kinase
siRNA: Small interfering ribonucleic acid
siCNS: siRNA control non-silencing
siILK/siITGA5/siITGAV: siRNA directed against ILK/ITGA5/ITGAV
TRITC: Tetramethylrhodamine
WB: Western blot
