# Supplementary material for: ILK supports RhoA/ROCK-mediated contractility of human intestinal epithelial crypt cells by inducing the fibrillogenesis of endogenous soluble fibronectin during the spreading process
Source: BMC Mol Cell Biol. 2020 Mar 17;21:14. doi: 10.1186/s12860-020-00259-0 (PMC7079544; doi:10.1186/s12860-020-00259-0)
Supplement: Supplementary file 1 — Additional file 1. ILK-depletion does not prevent tensin recruitment to peripheral adhesion sites in HIECs. (a) Western blot analysis shows comparable levels of tensin detected in siCNS and siILK cells. (b) Epifluorescence microscopy images representative of tensin distribution siCNS and siILK cells growth on pretreated and FN-coated (3 μg/cm2) glass coverslips. Adhesion to FN restores a fibrillar distribution of tensin in HIEC siILK cells. Scale bar in (b): 10 μm. [file 12860_2020_259_MOESM1_ESM.pdf]

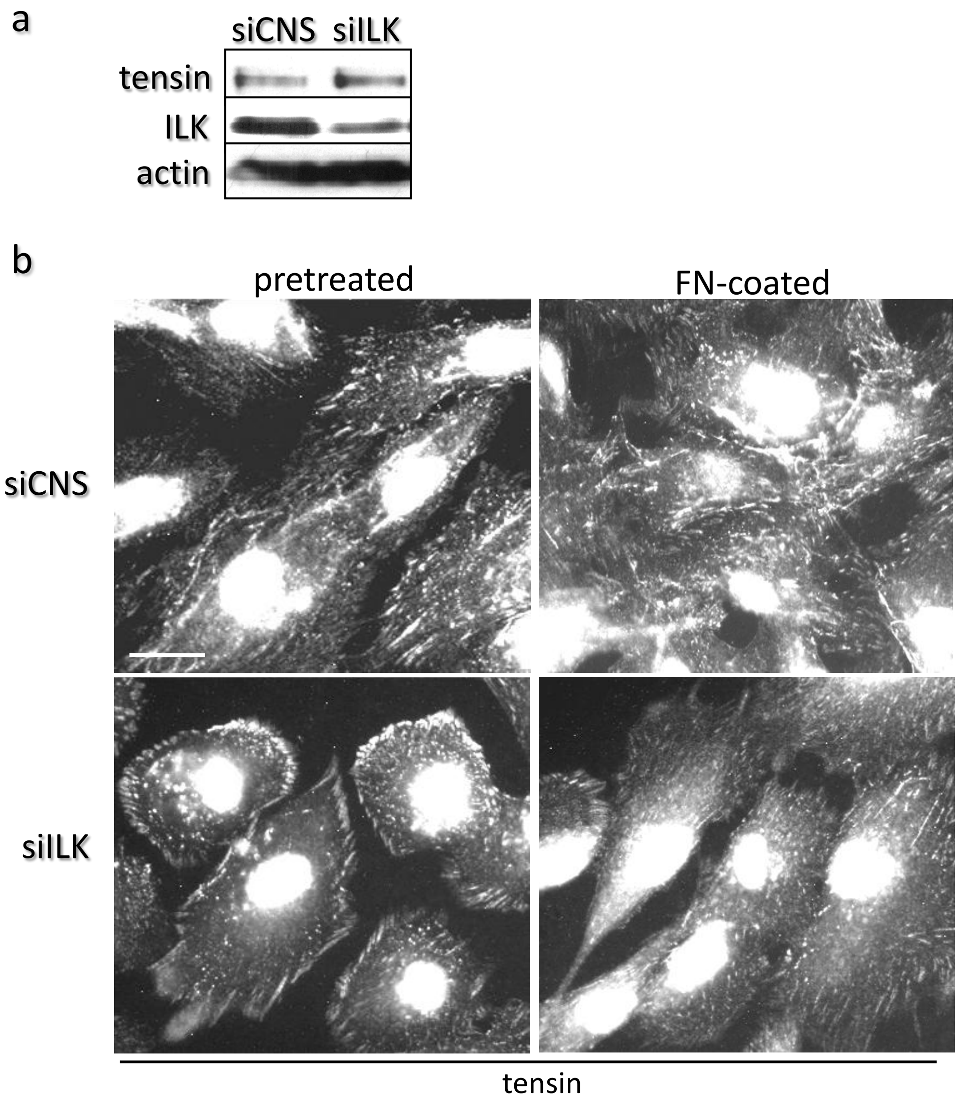

Additional file 1 - ILK-depletion does not prevent tensin recruitment to peripheral adhesion sites in HIECs. (a) Western blot analysis shows comparable levels of tensin detected in siCNS and siILK cells. (b) Epifluorescence microscopy images representative of tensin distribution siCNS and siILK cells growth on pretreated (uncoated) and FN-coated (3 µg/cm^2) glass coverslips. siILK cells adhesion to FN restores a fibrillar distribution of tensin out of the peripheral IACs. Scale bars in (b): 10 µm
