# Supplementary material for: ILK supports RhoA/ROCK-mediated contractility of human intestinal epithelial crypt cells by inducing the fibrillogenesis of endogenous soluble fibronectin during the spreading process
Source: BMC Mol Cell Biol. 2020 Mar 17;21:14. doi: 10.1186/s12860-020-00259-0 (PMC7079544; doi:10.1186/s12860-020-00259-0)
Supplement: Supplementary file 2 — Additional file 2. Adding exogenous soluble FN to the medium does not rescue the contractile phenotype in ILK-depleted HIECs. (a) Inverted contrast microscopy images of siCNS and siILK plated and grown for 24 h on uncoated plastic dishes before (upper panels) and 24 h after (lower panels) adding medium containing human plasma FN (17 μg/ml final concentration ≅ 3 μg/cm2). (b) The cells were plated on uncoated (upper panels) and FN-coated (3 μg/cm2; lower panels) dishes 4 h before adding human plasma FN to the medium and grown for an additional 24 h under these conditions. Scale bars in (a and b): 30 μm. [file 12860_2020_259_MOESM2_ESM.pdf]

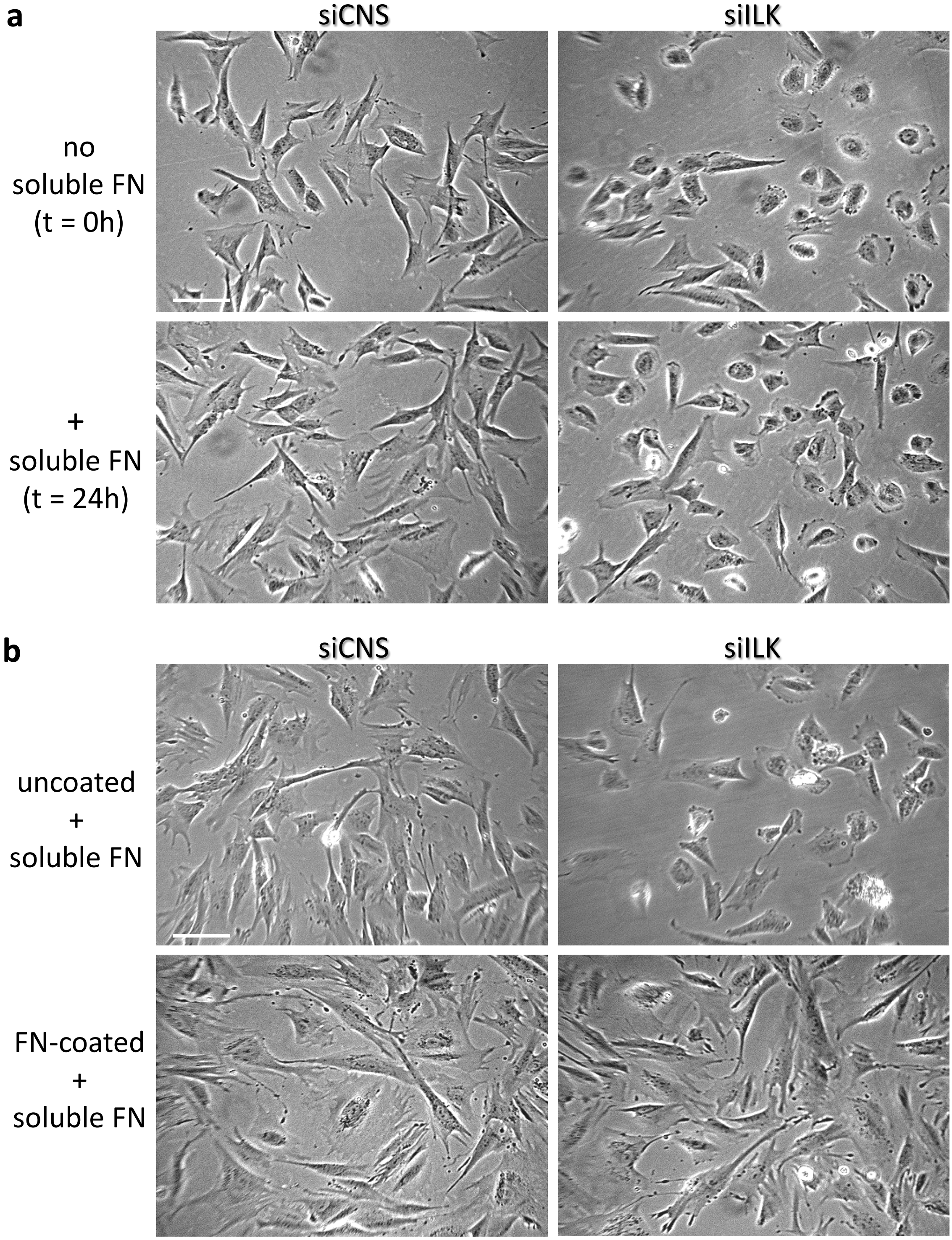


**Additional file 2 - Adding exogenous soluble FN to the medium does not rescue the contractile phenotype in ILK-depleted HIEC.** (a) Inverted contrast microscopy images of siCNS and siILK plated and grown for 24h on uncoated plastic dishes before (upper panels) and 24h after (lower panels) adding medium containing human plasma FN (17 ug/ml final concentration ~ 3 µg/cm$^2$). (b) The cells were plated on uncoated (upper panels) and FN-coated (3 µg/cm$^2$ ; lower panels) dishes 4h before adding human plasma FN to the medium and grown for an additional 24h under these conditions. Scale bars in (a and b): 30 µm.
